# Human α-Defensin Expression Is Not Dependent on CCAAT/Enhancer Binding Protein-ε in a Murine Model

**DOI:** 10.1371/journal.pone.0092471

**Published:** 2014-03-21

**Authors:** Andreas Glenthøj, Sara Dahl, Maria T. Larsen, Jack B. Cowland, Niels Borregaard

**Affiliations:** The Granulocyte Research Laboratory, Department of Hematology, National University Hospital, University of Copenhagen, Copenhagen, Denmark; University of Hyderabad, India

## Abstract

Specific granule deficiency (SGD) is a rare congenital disorder characterized by recurrent infections. The disease is caused by inactivating mutations of the CCAAT/enhancer binding protein-ε (C/EBP-ε) gene. As a consequence, specific and gelatinase granules lack most matrix proteins. Furthermore, azurophil granules contain diminished amounts of their most abundant proteins, α-defensins, also known as human neutrophil peptides (HNPs). In accordance with this, *in vitro* models have demonstrated induction of HNPs by C/EBP-ε. Since mice do not express myeloid defensins, they cannot per se be used to characterize the role of C/EBP-ε in controlling HNP expression *in vivo*. We therefore crossed a transgenic HNP-1-expressing mouse with the Cebpe^-/-^ mouse to study the *in vivo* significance of C/EBP-ε for HNP-1 transcription and expression. Surprisingly, neither expression nor processing of HNP-1 was affected by lack of C/EBP-ε in these mice. Transduction of C/EBP-ε into primary bone marrow cells from HNP-1 mice induced some HNP-1 expression, but not to levels comparable to expression human cells. Taken together, our data infer that the HNP-1 of the transgenic mouse does not show an expression pattern equivalent to endogenous secondary granule proteins. This limits the use of these transgenic mice as a model for human conditions.

## Introduction

Specific granule deficiency (SGD) is a rare congenital disorder caused by a defect in formation of peroxidase negative neutrophil granules. Clinically, the patients suffer from recurrent infections, often in the form of abscesses. Their number of neutrophils is generally within the normal range, but these are structurally characterized by the pseudo-Pelger-Huet nuclear abnormality and by lack or minimal levels of proteins localized to the matrix of peroxidase negative granules such as lactoferrin and vitamin-B_12_-binding protein [1]. Furthermore, their azurophil granules are lighter than normal [2] and contain little or none of the most abundant of azurophil granule proteins, human neutrophil peptides (HNPs), which constitute 30–50% of the azurophil granule content in neutrophil from healthy donors [3]. Functionally, the neutrophils are deficient in chemotaxis and have a reduced NADPH oxidase activity. The disorder is caused by mutations in the CCAAT/enhancer binding protein-ε (C/EBP-ε) [4], [5], a transcription factor essential for neutrophil development beyond the promyelocyte stage. C/EBP-ε is critical for transcription of most granule proteins localized to specific and gelatinase granules as well as for azurophil granule proteins expressed in the late promyelocyte stage such as bactericidal permeability increasing protein (BPI) and HNPs [1], [4], [6]. HNPs and BPI localize in a subset of azurophil granules and are largely regulated similarly to specific granule proteins (SGPs) with peak transcription in myelocytes/metamyelocytes [7], [8] and are strongly induced by C/EBP-ε *in vitro* [9], [10]. In accordance with this, HNPs are reduced by over 90% in SGD [11].

Four isoforms of C/EBP-ε have been described in humans (32, 30, 27, and 14 kDa), but their function seem to differ. The 32/30 kDa isoforms are transcriptional activators, whereas the 27 and 14 kDa isoforms have been suggested to function as repressors of GATA-1 and C/EBPs respectively [12]. The 32/30 kDa isoforms are relatively weak transactivators and require co-activators such as c-myb for optimal function [13]. In contrast, mice only generate one C/EBP-ε mRNA transcript, which can give rise to a 36 and 34 kDa isoform through use of alternative translational start sites [14], [15].

HNPs are small cationic peptides with broad antimicrobial properties. They are synthesized as inert and non-polar proHNPs [16], which are processed by (an) unidentified protease(s) to cationic HNPs in promyelocytes and are retained intracellularly via binding to the negatively charged proteoglycan serglycin [17]. In myelocytes and metamyelocytes that produce large amount of proHNP, the proform is not cleaved and most is secreted into the bone marrow (BM) plasma [7], [18]. It has not yet been examined, whether the reduced amounts of HNPs in SGD are merely a result of reduced transcription of HNPs or whether the posttranslational processing and cellular retention of HNPs might also be impaired by lack of C/EBP-ε.

The Cebpe^-/-^ mouse is an excellent model of SGD, but since mice do not express myeloid defensins [19], this model cannot be used directly to characterize the role of C/EBP-ε in controlling HNP expression *in vivo*. We therefore crossed the transgenic HNP-1 mouse [20] with the Cebpe^-/-^ mouse to study the *in vivo* significance of C/EBP-ε for HNP-1 transcription and processing. Neutrophils from the transgenic HNP-1 mouse contain less than 10% of the HNP-1 present in human neutrophils [20]. This obviously limits the usefulness of the HNP-1 transgene as a mouse model for studying the role of HNP in innate immunity, but the model can be useful for studying regulatory aspects of HNP-1 expression. Myeloid α-defensin genes are subject to extensive copy number variations ranging from 2 to 22 *DEFA1/DEFA3* genes per diploid genome [21]–[24], and neutrophil α-defensin content has been positively related to copy number [21]. With approximately 80 copies of full length *DEFA1* integrated into the transgenic HNP-1 mouse genome [20], a high expression of HNP-1 in neutrophils would be expected, and the reason for their low content is unknown. Mice transgenic for α-defensins not dependent on C/EBP-ε, e.g. the enteric human defensin 5 or 6, have shown expression levels comparable to human conditions [25], [26] as mice naturally express enteric α-defensins.

An explanation for the low HNP-1 expression in the transgenic HNP-1 mouse could be lack of responsiveness to murine C/EBP-ε. To test this, we transduced human C/EBP-ε into primary bone marrow cells of the transgenic HNP-1 mouse.

## Materials and Methods

### Ethics statement

Animal breeding and experiments were performed according to permission (#2006/562−43) and guidelines from the Danish Animal Experiments Agency. Human BM aspirates were obtained after informed written consent according to the permission (H-1-2011-165) and guidelines from the ethics committee of the Capital Region of Denmark.

### Statistical analyses

Statistical calculations were performed with Graphpad 5.0 (Graphpad Software Inc.). Tests were two-tailed and the significance level was set to P<0.05. Number of experiments is stated in figure legends.

### Real-time quantitative PCR

RNA isolation and cDNA synthesis were performed as previously described [27]. cDNA was subjected to real-time quantitative polymerase chain reaction (PCR) analysis using TaqMan gene expression assays (Applied Biosystems) on a 7500 Real-Time PCR system, according to the manufacturer's instructions. Assays included: *DEFA1* [17] for measurement of human HNP-1 in transgenic mice (Hs00234383_m1) and the murine markers myeloperoxidase (*Mpo*; Mm00447875_g1), lipocalin-2 (*Lcn2*; Mm00809552_s1), cathelin-related antimicrobial peptide (*Camp*; Mm00438285_m1), lactoferrin (*Ltf*; Mm00434787_m1), and matrix metalloproteinase-9 (*Mmp9*; Mm00600164_g1). Expression levels were normalized to the constitutively expressed murine housekeeping gene *Gapdh* (4352339E).

### Antibodies

The following antibodies were used: rabbit anti-proHNP [28], rabbit anti-HNP [29], rabbit anti-GAPDH (2118; Cell Signaling Technology), rabbit control IgG (X0903; Dako), C/EBP-ε (sc -158x; Santa Cruz Biotech), 24p3 (AF1857; R&D Systems), beta-actin (sc-1616, Santa Cruz Biotech), biotin rat anti-mouse IgG2b (553987; BD Biosciences), and biotin rat anti-mouse CD11b (51-01712J; BD Biosciences).

### Western blotting

SDS-Tricine-PAGE [30] and immunoblotting [31] were performed as previously described [17].

### Pulse-chase biosynthesis

Pulse-chase biosynthesis was performed as previously described [17].

### Immunocytochemistry

Immunocytochemistry was performed as previously described [17].

### Mice

C57BL/6 was used as background strain and mice were backcrossed for >10 generations. Frozen embryos from the transgenic HNP-1 mouse [20] were obtained from ATCC with the kind permission of Dr. Rose Linzmeier, David Geffen School of Medicine, UCLA. Cebpe^-/-^ mice [14] were a kind gift from Dr. Adrian F. Gombart, Linus Pauling Institute, Oregon State University. Genotyping was done on DNA from tail tips using primers as previously described [17]. HNP-1 gene copies in mice were determined by real-time quantitative PCR on tail DNA using a TaqMan Custom gene expression assays specific to the genomic sequence for HNP-1 (*DEFA1*).

### Isolation of BM cells

Murine BM cells were isolated as previously described [17]. Where indicated, cells were depleted of non-granulocytic cells by immunomagnetic sorting using biotinylated antibodies against surface epitopes of T-cells (CD3e; 51-01082J), B-cells (CD45r; 51-01122J), and erythroid cells (TER-119; 51-09082J), (all BD Bioscience) and the magnetic cell sorting (MACS) system according to instructions of the manufacturer (Miltenyi). Human BM aspirates were depleted of erythrocytes by dextran sedimentation and hypotonic lysis.

### Chromatin Immunoprecipitation (ChIP)

ChIP assays were performed using Magna ChIP A/G Chromatin Immunoprecipitation Kit (Millipore) according to the manufacturer's instructions. Briefly, 1.5×10^7^ bone marrow cells were cross-linked by 1% formaldehyde, washed in phosphate-buffered saline (PBS), and incubated in 750 μL of cell lysis buffer for 15 minutes. Lysed cells were spun down and the pellet resuspended in 750 μL nuclear lysis buffer and sonicated (10 pulses of 10 microns, 10 seconds each) in a Soniprep 150 sonicator (Sanyo). Insoluble material was removed by centrifugation and aliquots of 50 μL supernatant were used for immunoprecipitation. Efficient breakdown of chromatin by sonication was verified by electrophoresis. Chromatin was immunoprecipitated using antibodies against C/EBP-ε (4 μg, sc-158; Santa Cruz Biotech) or rabbit IgG (4 μg, X090; Dako) and protein A/G magnetic beads overnight at 4°C with rotation. The following day, precipitates were washed, immune complexes eluted and reversed, and DNA was recovered with the supplied spin columns. ChIP DNA was used as a template for quantitative PCR using Power SYBR Green (Applied Biosystems) on a 7500 Real-Time PCR system, according to the manufacturer's instructions. The lengths of amplicons were checked on agarose gels. The following primers were used: HNP C/EBP: forward primer (FP), 5′-GTCAACTGTGTTAGGAGCCAT-3′; reverse primer (RP), 5′-CGTGCACAAGTGGACTTC-3′. Murine Camp C/EBP: FP, 5′-GATAGTCCCTCTGGGGCC-3′; RP, 5′-GAGCCTCATTTATTCTCATCC-3′. Murine control: FP, 5′-ACCAGGGAGGGCTGCAGTCC-3′; RP, 5′-TCAGTTCGGAGCCCACACGC-3′. Human CAMP C/EBP: FP, 5′-CGTGCCCTGCCTCATTC-3′; RP, 5′-TGGTCCCCATGTCTGCC-3′. Human control: FP, 5′-ATGGTTGCCACTGGGGATCT-3′; RP, 5′-TGCCAAAGCCTAGGGGAAGA-3′.

### Retroviral transduction

pMIG-CEBPE [32] containing the coding sequence of human 32 kDa C/EBP-ε inserted upstream of IRES-GFP allowing for co-expression of C/EBP-ε and green fluorescent protein (GFP) was kindly donated by Dr. Philip Koeffler. A similar vector, pMIG-Cebpe, expressing the coding sequence of murine C/EBP-ε was created as previously described [33]. The Phoenix E packaging cell line [34] was transiently transfected with a retroviral vector (pMIG, pMIG-CEBPE, or pMIG-Cebpe) using the calcium phosphate chloroquin method along with the pCl-ECO packaging vector to obtain higher retroviral titers. Cellular supernatant containing retrovirus was harvested two days later and filtered through a 0.45-μm filter. Murine BM cells were isolated and separated on discontinuous 1.072 Percoll/PBS (GE Healthcare) gradient as previously described [17]. Interphase cells were washed, resuspended in Iscove's Modified Dulbecco's Medium (IMDM) with Glutamax, 15% FCS +100 U/mL penicillin, 100 μg/mL streptomycin (all from Invitrogen), 100 ng/ml murine G-CSF (PeproTech), and 10 ng/ml murin IL-3 (Sigma), and placed in a humidified incubator with 5% CO_2_ at 37°C. Cells were transduced twice using Retronectin (CH-296; Takara) coated wells and protamin sulfate for enhanced transduction efficiency. Cells were harvested for further analysis 42 hours after the last transduction. Transduction efficiency was determined on a FACSCalibur (BD Bioscience) as percentage GFP-positive cells. 10^5^ cells were labeled with biotinylated rat anti-mouse CD11b antibody (51-01712J; BD Biosciences).

## Results

### HNP-1 expression is not affected by lack of C/EBP-ε in mice

**Figure 1:**
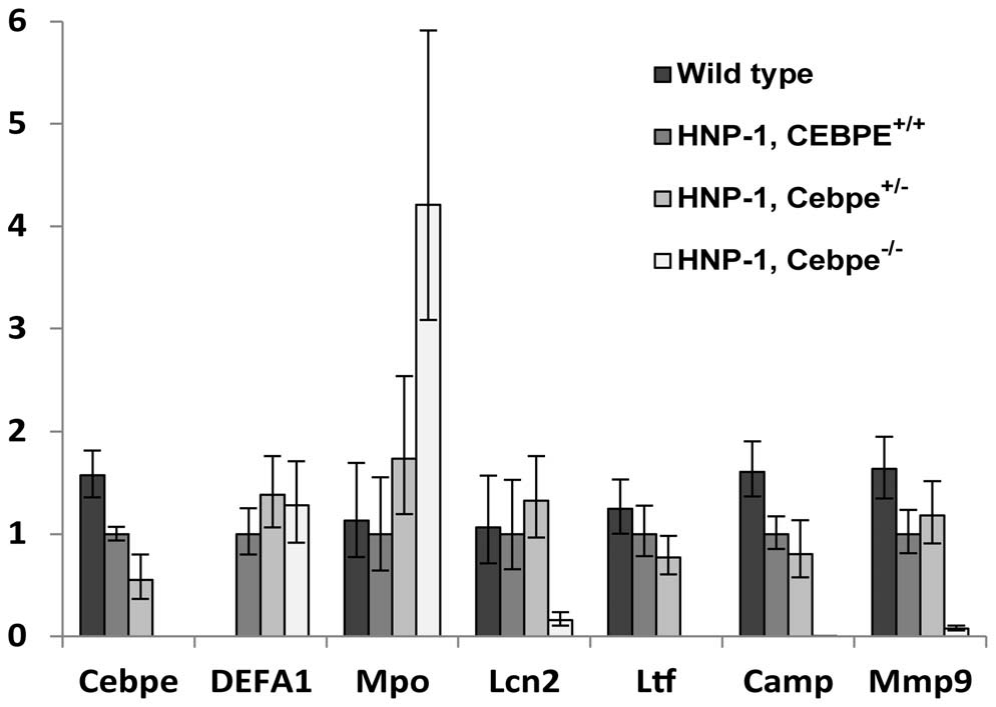
Characterization of granulocytic bone marrow cells of transgenic HNP-1 mice deficient in C/EBP-ε. Murine bone marrow cells were extracted from wild type and transgenic HNP-1 mice with or without deficiency in C/EBP-ε. Erythrocytes and non-granulocytic cells removed by ammonium chloride-based lysis and by immunomagnetic sorting, respectively. Comparative quantification of mRNA for CCAAT/enhancer binding protein-ε (*Cebpe*), human neutrophil peptide-1 (*DEFA1*), myeloperoxidase (*Mpo*), 24p3 (*Lcn2*), lactoferrin (*Ltf*), cathelicidin antimicrobial peptide (*Camp*), matrix metalloproteinase-9 (*Mmp9*) was done by real-time PCR using *Gapdh* as normalizer. Figure depicts expression levels relative to HNP-1, Cebpe^+/+^ mice. Error bars were calculated by Stratagene MxPro 4.1. Data are representative of two independent experiments.

**Figure 2:**
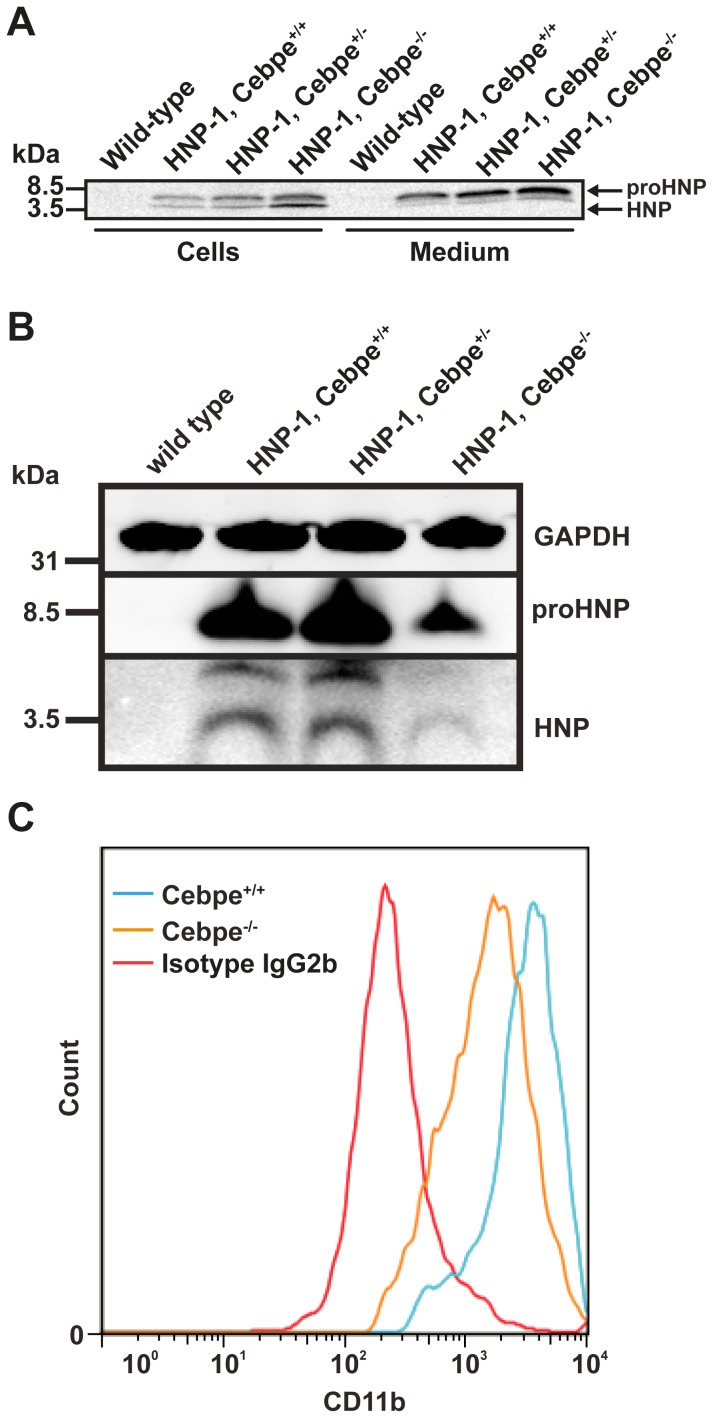
Biosynthesis and Western blotting of proHNP and HNP in HNP-1 mice deficient in C/EBP-ε. Murine bone marrow (BM) cells from one wild type and transgenic HNP-1 mice with or without deficiency in C/EBP-ε were extracted. (A) Cells were pulsed with ^35^S-methionine/cysteine for 1 hour and chased for 5 hours. Cell lysates and medium were immunoprecipitated with antibodies against proHNP and HNP. Immunoprecipitates were pooled and analyzed by 16% SDS-Tricine-PAGE and fluorography. (B) Non-granulocytic cells from BM of wild type mice, transgenic HNP-1 mice (HNP-1, Cebpe^+/+^), and HNP-1 mice heterozygous or deficient for C/EBP-ε (HNP-1, Cebpe^+/−^ or Cebpe^-/-^) were removed by ammonium chloride-based lysis and by immunomagnetic sorting. Western blotting was performed for HNP, proHNP, and GAPDH and is representative of two independent experiments. (C) BM cells from Cebpe^+/+^ (blue) or Cebpe^-/-^mice (orange) were immunomagnetically depleted of non-granulocytic cells and labeled with biotinylated rat anti-mouse CD11b antibody and analyzed by flow cytometry using biotinylated rat anti-mouse IgG2b as isotype control. Data are representative of four independent experiments.

Transgenic HNP-1 mice were crossbred with Cebpe^-/-^ mice to test *in vivo* the significance of C/EBP-ε for HNP-1 expression. Mice were euthanized, bone marrow (BM) cells extracted, and granulocytic precursors purified by immunomagnetic sorting. As expected from previous studies [35], BM cells from Cebpe^-/-^ mice were deficient in transcription of the SGPs *Ltf* and *Camp* (Figure 1). As described before [36], *Mpo* transcription was higher as an indicator of the relatively increased amount of immature granulocytic precursors present in the BM of Cebpe^-/-^ mice (Figure 1 and 2A-D). In agreement with earlier findings [36], [37], the SGP 24p3 (Lcn2), the murine orthologue of human neutrophil gelatinase-associated lipocalin (NGAL), as well as the gelatinase granule protein matrix metalloproteinase-9 (Mmp9), showed some residual expression in Cebpe ^-/-^ mice. Surprisingly, *DEFA1* expression was not diminished by lack of C/EBP-ε in the transgenic mouse.

### Post-tranlational processing of HNP-1 is not inhibited by lack of C/EBP-ε in mice

**Figure 3:**
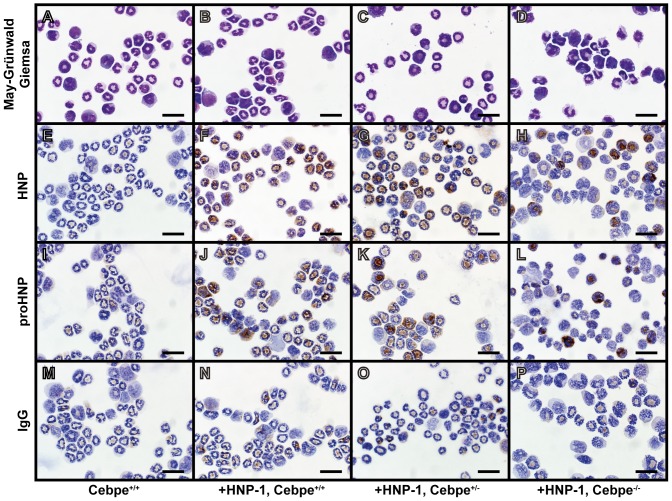
Immunocytochemical staining of murine bone marrow cells from wild type and HNP-1 mice. Murine bone marrow from wild type and transgenic HNP-1 mice with or without deficiency in C/EBP-ε was extracted. Non-granulocytic cells were removed by ammonium chloride-based lysis and by immunomagnetic sorting. Cells were spun onto slides, fixed, permeabilized, and stained: (A-D) May-Grünwald Giemsa, (E-H) HNP, (I-L) proHNP, and (M-P) IgG control. Bars represent 20 μm.

We next tested whether the posttranslational processing of HNP-1 might be affected in Cebpe^-/-^ mice by pulse-chase biosynthesis on BM cells from HNP-1, Cebpe^-/-^ mice (Figure 2A). DEFA1, Cebpe^-/-^ mice did not show any deficiency in expression nor processing of proHNP. On the contrary, expression seemed a bit higher in the DEFA1, Cebpe^-/-^ mice. Immunocytochemistry and Western blotting of murine BM cells confirmed the intact ability of Cebpe^-/-^ mice to transcribe and store HNP-1 (Figure 2 and 3E-L). Despite unaltered expression of *DEFA1* mRNA in transgenic HNP-1 mice deficient in C/EBP-ε (Figure 1), Western blotting showed significant reduction of proHNP and HNP in DEFA1, Cebpe^-/-^ mice compared to their C/EBP-ε^+/+^ counterparts (Figure 2A-B). The neutrophil differentiation marker CD11b was less prevalent in BM cells of the Cebpe^-/-^ mice (Figure 2C) demonstrating the more immature phenotype of the neutrophil precursors in these animals.

### Murine C/EBP-ε binds the HNP-1 promoter *in vivo*

**Figure 4:**
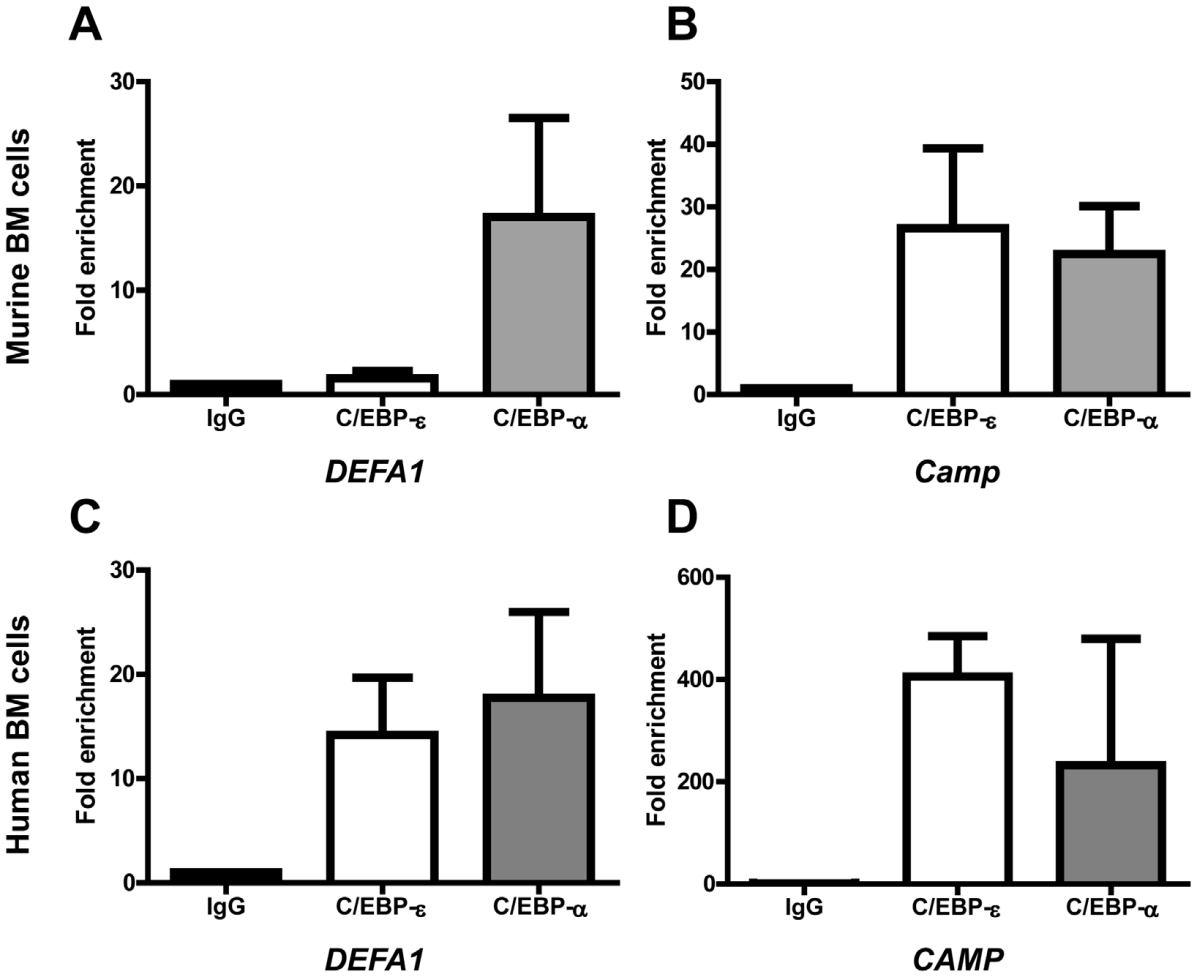
Chromatin immunoprecipitation (ChIP) analysis of the HNP-1 promoter in murine and human bone marrow (BM) cells. BM cells from 3 mice (A–B) and two healthy human donors (C–D) were extracted and fixed in formaldehyde. Cell and nuclear membranes were lysed before fragmentation of DNA by sonication. Chromatin was immunoprecipitated using protein A/G magnetic beads and an antibody against C/EBP-ε, C/EBP-α, or negative control rabbit IgG. After washing procedures, immune complexes were eluted and reversed and DNA recovered. DNA was used as a template for quantitative PCR. Primers used were specific for putative C/EBP sites in the (A,C) HNP-1 promoter and (B,D) murine and human promoters of the specific granule protein cathelin-related antimicrobial peptide (CAMP). Levels are depicted as fold enrichment compared to negative control IgG immunoprecipitation. Error bars depict standard deviation.

We performed chromatin immunoprecipitation (ChIP) to investigate whether murine C/EBP-ε is able to bind to CCAAT sites in the *DEFA1* promoter. Bone marrow cells from transgenic HNP-1 mice were depleted of non-granulocytic cells and cross-linked with formaldehyde. Chromatin was immunoprecipitated with antibodies against C/EBP-ε and C/EBP-α and binding to promoter regions was probed using PCR-reactions specific for granule protein gene promoters. Murine C/EBP-ε and C/EBP-α bound the promoter of the SGP cathelin-related antimicrobial peptide (*Camp*) as well as the *DEFA1* promoter, although the signal of C/EBP-ε binding to the *DEFA1* promoter indicated significantly less binding than to the *Camp* promoter (Figure 4A–B).

So far, binding of human C/EBP-ε to the *DEFA1* promoter has only been demonstrated in cell lines[38]. To assure that this is also the case *in vivo*, we performed ChIP on primary human bone marrow precursors. As expected, quantitative PCR of precipitated DNA showed excellent binding of human C/EBP-ε to the *DEFA1* as well as the *CAMP* promoter (Figure 4C–D).

### Overexpression of C/EBP-ε can induce expression of transgenic HNP-1

**Figure 5:**
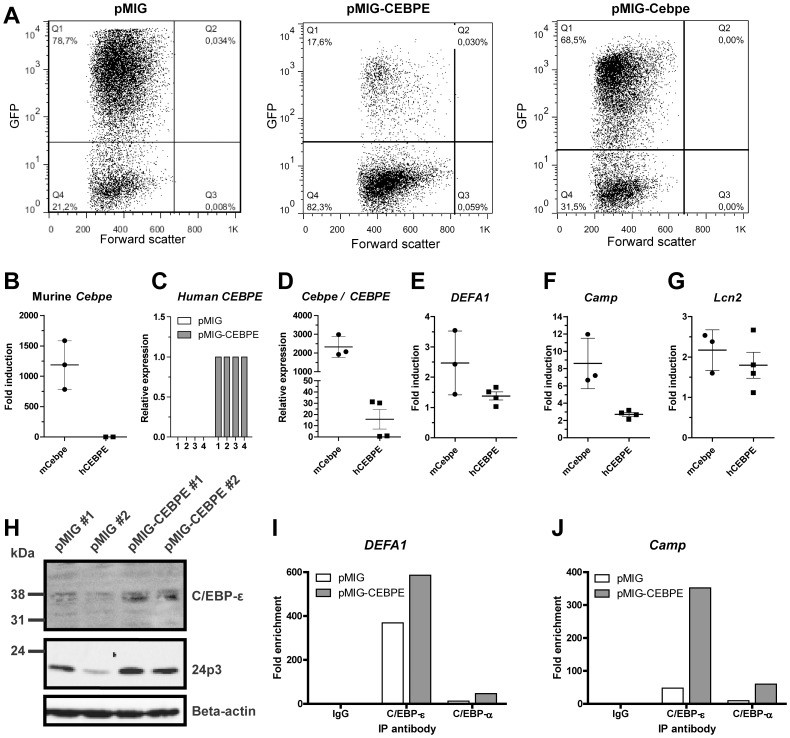
Human and murine C/EBP-ε induces expression of HNP-1 in primary bone marrow (BM) cells from transgenic HNP-1 mice. Murine BM cells from seven transgenic HNP-1 mice were isolated and early granulocyte precursors isolated by density centrifugation on a discontinuous Percoll 1.072 gradient. Cells were retrovirally transduced with an empty expression vector (pMIG) or with a vector expressing either human or murine C/EBP-ε (pMIG-CEBPE or pMIG-Cebpe respectively). Cells were incubated for 48 hours. (A) Green fluorescent protein (GFP) was used as reporter gene in the vectors and transduction efficiency evaluated by flow cytometry. (B–G) Comparative quantification of mRNA for CCAAT/enhancer binding protein-ε (human *CEBPE* or murine *Cebpe*), human neutrophil peptide-1 (*DEFA1*), cathelicidin antimicrobial peptide (*Camp*), and lipocalin-2 (*Lcn2*) was done by real-time PCR using *Gapdh* as normalizer. Error bars depict standard deviation. (B, E–G) Levels are shown as fold induction by either murine *Cebpe* (mCebpe) or human *CEBPE* (hCEBPE) compared to levels from negative control transduction (pMIG). (C) Relative quantification of human *CEBPE* in murine bone marrow cells from four transgenic HNP-1 mice transduced with control vector (pMIG) or human *CEBPE*. (D) Expression of murine *Cebpe* in *Cebpe* transduced cells were compared to human *CEBPE* in *CEBPE* transduced cells by comparing Delta Ct between the transduced gene and *Gapdh*. The transduced mouse with the lowest expression of C/EBP-ε was used as calibrator. (H) Western blotting of C/EBP-ε, 24p3, and beta-actin in transduced cells from two mice. (I–J) Cells were fixed in formaldehyde. Cell and nuclear membranes were lysed before fragmentation of DNA by sonication. Chromatin was immunoprecipitated using protein A/G magnetic beads and an antibody against C/EBP-ε, C/EBP-α, or negative control rabbit IgG. After washing procedures, immune complexes were eluted and reversed and DNA recovered. DNA was used as a template for quantitative PCR. Primers used were specific for putative C/EBP sites in the *DEFA1* promoter and promoters of the specific granule protein cathelin-related antimicrobial peptide (*Camp*). Levels are depicted as fold enrichment compared to negative control IgG immunoprecipitation.

Next, we examined whether transduction with human or murine C/EBP-ε might induce expression of transgenic HNP-1. Bone marrow cells from transgenic HNP-1 mice were isolated and immature granulocyte precursors isolated by density centrifugation. Cells were retrovirally transduced with the pMIG expression vector - either empty or containing the coding sequence of full length human or murine C/EBP-ε. Transduction efficiency was 50.0-82,3% for pMIG control, 66,1–74,3% for murine pMIG-Cebpe, and 16.6–17,7% for human pMIG-CEBPE (Figure 5A). Murine *Cebpe* was induced >1000-fold in cells transduced with murine C/EBP-ε (Figure 5B). Human C/EBP-ε was also successfully transduced into the murine cells (Figure 5C). However, when compared to endogenous *Gapdh*, transduction of murine *Cebpe* appeared many-fold more efficient than its human counterpart, *CEBPE* (Figure 5D). Expression of *DEFA1*, *Camp*, and *Lcn2* were induced by 1.38, 2.71, and 1.80-fold, respectively, in cells transduced with human C/EBP- ε compared to cells transduced with the empty vector (Figure 5E–G), whereas transduction of murine *Cebpe* provided a fold induction of 2.47, 8.60, and 2.17, respectively. Human C/EBP- ε was detected in transduced cells by Western blotting (Figure 5H). The amount of the SGP, 24p3, was approximately doubled in cells transduced with human C/EBP-ε (Figure 5I). Binding of C/EBP-ε to the *DEFA1* and *Camp* promoter was demonstrated by ChIP (Figure 5J). Notably, binding of murine C/EBP-ε to the *DEFA1* promoter seemed more prominent in density separated cells (Figure 5J) than in cells immunomagnetically depleted of T-, B-, and erythroid cells (Figure 4A) as the former is enriched in myelocytes and metamyelocytes where the level of C/EBP-ε peaks. Our antibody did not allow definitive discrimination between the human and murine C/EBP-ε orthologues.

## Discussion

C/EBP-ε is a key inducer of HNP expression. Although C/EBP-α can bind the *DEFA1* promoter as previously demonstrated in the human promyelocytic cell line NB4, it is fully displaced by C/EBP-ε upon induction of differentiation with ATRA [39]. This displacement is thought to be the initiating event for induction of the high HNP-1 expression seen in late promyelocytes and myelocytes. Our data show that human as well as murine C/EBP-ε binds the *DEFA1* promoter *in vivo*. Contrary to findings in SGD [1], *DEFA1* expression was not diminished by lack of C/EBP-ε in the transgenic mouse as seen by real-time quantitative PCR. This indicates that transcription of *DEFA1* is not dependent on murine C/EBP-ε in the transgenic HNP-1 mouse. C/EBP-ε expression in humans is more diverse with four isoforms with distinct functions [12], whereas mice only have two [14], [15]. Failure of murine C/EBP-ε to induce *DEFA1* expression could explain why the levels of HNP-1 in neutrophils of the transgenic mouse are in line with those of SGD neutrophils.

We transduced full length human and murine C/EBP-ε into the transgenic mouse BM cells. This induced *DEFA1* expression by 38% and 170% respectively. These low figures must be seen in the context of a transduction efficiency of only 17% for human *CEBPE* and 70% for murine *Cebpe*, indicating that the induction in transduced cells is similar at 220% and 240%, respectively. Although far from reaching the levels in human neutrophils, this indicates that the human *DEFA1* promoter present in the transgene is somewhat responsive to overexpression of C/EBP-ε. It is possible that co-activators required for full activation of *DEFA1* by C/EBP-ε are lacking in mice. Or, as seen in other models using of concatemeric transgenic inserts [40], [41], *DEFA1* could be subjected to epigenetic silencing modifications such as DNA methylation or histone modifications. Treatment with histone deacetylase inhibitors might alleviate this inhibition [42]. The integration site itself is another important factor which determines transgenic gene expression. Such positions effects are in general suppressive and more prominent if multiple nearly identical transcription factors are juxtaposed [43]. Such phenomena may very well influence HNP-1 expression in the transgenic mouse in which approximately 20 bacterial artificial chromosome (BAC) inserts, each containing 4 copies of *DEFA1* as well as a truncated version of the *DEFA3* gene, are integrated into a single chromosomal site. We have previously shown that unprocessed proHNP is primarily secreted into the bone marrow plasma, whereas fully processed HNP is retained in azurophil granules [17]. Diminished posttranslational cleavage of proHNP could thus contribute to the shortage of HNPs observed in neutrophils of SGD patients. The enzymes responsible for posttranslational processing of proHNP are still not known and it is possible that these might be expressed under control of C/EBP-ε albeit processing ceases when C/EBP-ε expression is at its peak [44]. We found intact posttranslational processing of HNP-1 by pulse-chase biosynthesis, but diminished amounts by Western blotting. In line with earlier findings [14], [45], BM cells of the Cebpe^-/-^ mice expressed less CD11b reflecting a lower degree of neutrophil precursors differentiation. Taken together, this implies that although expression of *DEFA1* is not affected by the lack of C/EBP-ε, the mere lack of maturation beyond the promyelocytic stage gives the mice a significantly shorter timeframe to synthesize and store HNPs. This could also contribute to the reduction of HNPs in SGD, although the pivotal decrease in *DEFA1* transcription in this setting is presumably the major factor responsible.

Taken together, our data infer that HNP-1, which lacks a murine orthologue, introduced into a murine model do not show an expression pattern equivalent to endogenous SGPs and limits the use of these mice as a model for human conditions.
